# Advanced Large-Scale Nanofabrication Route for Ultrasensitive SERS Platforms Based on Precisely Shaped Gold Nanostructures

**DOI:** 10.3390/nano11071806

**Published:** 2021-07-12

**Authors:** Suzanna Akil, Rana Omar, Dmitry Kuznetsov, Vladimir Shur, Aotmane En Naciri, Safi Jradi

**Affiliations:** 1 LCP-A2MC, Jean Barriol Institute, Lorraine University, 1 Arago Avenue, 57070 Metz, France; rana.omar@univ-lorraine.fr (R.O.); aotmane.en-naciri@univ-lorraine.fr (A.E.N.); 2 School of Natural Sciences and Mathematics, Ural Federal University, 51 Lenin Avenue, 620000 Ekaterinburg, Russia; dimak@urfu.ru (D.K.); vladimir.shur@urfu.ru (V.S.); 3 Light, Nanomaterials, Nanotechnologies (L2n) Laboratory, CNRS ERL 7004, University of Technology of Troyes, 12 Rue Marie Curie, 10004 Troyes, France; safi.jradi@utt.fr

**Keywords:** nanofabrication, self-assembly, large area, advanced nanocomposites, sensors

## Abstract

One of the key issues for SERS-based trace applications is engineering structurally uniform substrates with ultrasensitivity, stability, and good reproducibility. A label-free, cost-effective, and reproducible fabrication strategy of ultrasensitive SERS sensors was reported in this work. Herein, we present recent progress in self-assembly-based synthesis to elaborate precisely shaped and abundant gold nanoparticles in a large area. We demonstrated that shape control is driven by the selective adsorption of a cation (Na^+^, K^+^, and H^+^) on a single facet of gold nanocrystal seeds during the growth process. We studied SERS features as a function of morphology. Importantly, we found a correlation between the shape and experimental SERS enhancement factors. We observed a detection threshold of 10^−20^ M of bipyridine ethylene (BPE), which matches the lowest value determined in literature for BPE until now. Such novel sensing finding could be very promising for diseases and pathogen detection and opens up an avenue toward predicting which other morphologies could offer improved sensitivity.

## 1. Introduction

As the restoring force on the conduction electrons is extraordinarily sensitive to the particle curvature, non-spherical metallic nanoparticles (MNPs) produced via various approaches [1,2,3] have been found to shift their surface plasmon frequency drastically, thus making them useful as multicolor diagnostic labels and for other optical devices [4]. Recent studies showed that turnover rates and selectivity of catalytic reactions can be strongly influenced by the shape of the NPs since their surface structures and active sites are tailored at the molecular level. [5]

By means of versatile colloidal methods, a variety of shapes have also been reported so far, including the conventional 3D shapes (cube, tetrahedron, octahedron, decahedron, and icosahedrons) and 1D or 2D shapes (rod, wire, plate, disc, triangle, hexagon, tetrapods, bi-pyramid, and highly branched structures) [6,7,8,9]. Moreover, the cubical shape is the most well-known in terms of a stable anisotropic morphology in colloidal methods.

The formation of different shapes is generally achieved by changing the reducing and stabilizing factors upon colloidal synthesis. In parallel, many studies have investigated the role of ligands (e.g., halides or small molecules such as citrate and cetyltrimethylammonium bromide) in controlling the NP morphology [10]. In addition to colloidal approaches, new methods have been proposed to synthesize non-spherical NPs. Abid et al. showed that silver NPs can be synthesized by various irradiation methods. Laser irradiation of aqueous solution of a silver salt and surfactant yields silver NPs with suitable shapes and sizes [11]. This corresponds to the photochemical reduction process that enables the shape evolution depending on the photonic parameters (light power, exposure time, etc.). Recently, an approach of DNA-mediated growth was reported where the morphological evolution of gold nano-prism seeds into different shapes takes place [12]. However, different physical, chemical, and biological methods have synthesized MNPs (Au, Pt, Cu, Al, etc.) of different shapes [13,14,15,16,17,18].

Among the colloidal synthetic strategies, Seed-mediated growth (SMG) is the most used one to fabricate MNPs of diverse shapes [15,19,20,21]. In this context, the growth mechanism of different morphologies of MNPs can be achieved through modifying the reaction conditions such as the surfactant chain length and concentration, the metal salt concentration as well the reduction kinetics (reducing agents and acid concentration), thus allowing the nanorods formation [15,20,21]. According to the SMG mechanism, the rate of the reaction (kinetics) is a clever combination of the four components cited above and could be a crucial factor in controlling the final shape of the NPs.

On the other hand, several additives could also play a significant role in determining the shape of MNPs in colloidal synthesis. Additives may correspond to surface factor rather to kinetic effects, which is induced by adjusting the solution composition. Various structure directing agents, including complexes (W(CO)_6_), reactive gas molecules (O_2_, H_2_, NO, etc.), and metal ions (Ag^+^, Cu^2+^, Fe^3+^, and Co^+^), can be used [18] [22]. Since El-Sayed’s group has demonstrated shape controlling of Platinum NPs (PtNPs) toward various shapes (cube, tetrahedron, octahedron, etc.), many studies explored the role of surfactants in shape-controlled synthesis of MNPs [23]. The idea was to control the surface energy of crystal facets of metal nanocrystals to produce a specific shape by adjusting the interactions of metal surfaces and capping agents. Therefore, the selection of the capping agent, its metal composition, and solution composition is crucial. As an example, PVP can interact strongly with the {100} direction of metal atoms, which changes both the surface free energies of the crystal facets and the relative growth rates. As a result, the binding of PVP to {100} facets of metal atoms results in a slower growth rate and then to the formation of triangles, tetrahedron, and octahedron shapes. Nevertheless, the bounding of PVP of random chains to {100} facets results in a fast-kinetic reaction, which results in a higher growth rate and gives rise to the formation of nanocubes [24,25].

To meet the challenge of shape-controlled synthesis, this paper attempts to fabricate GNPs with different shapes based on a previous work that used vapor induced phase separation (VIPS) strategy [26]. Thus, we aim to show the evolution of the cubic shape into another morphology by changing the major process components. After synthesis, the SERS features of the various morphologies were investigated to first evaluate their sensitivities. Then, the influence of shape on SERS features was investigated. Such a study is usually not simply achieved except for single NPs. This is because existing synthesis strategies do not permit a good control of dimensions, morphologies, and gaps between NPs simultaneously, as in our case. Here, we show an excellent control of these structural parameters, which allowed us to obtain a good understanding of shape impact in sensing for MNPs.

## 2. Materials and Methods

Synthesis method: In a previous study [26], the synthesis of monodisperse gold nanocubes was achieved by the combination of five components including acetone as a solvent for PMMA (Sigma-Aldrich, Kappelweg 1, Schnelldorf, Germany), ethanol (Sigma-Aldrich, Saint Louis, MO, USA) as a solvent of gold precursor, NaAuCl_4_ (Sigma-Aldrich, Saint Louis, MO, USA) (Sodium tetrachloroaurate) as the metal precursor, high concentrations of gold precursor, and a high evaporation rate of solvents. In order to change the shape from cubic, we fixed the conditions for all samples: (i) high gold precursor concentration (100 mM), (ii) 5000 rpm as spin-coating speed, and (iii) the silicon wafer (Arsenic-doped). We varied the solution composition to change the final shape of GNPs. We performed several combinations of PMMA/gold precursor dispersion to vary the growth rate of GNPs. Thus, all three main parameters are the following: the precursor molecule and the solvent of both PMMA and gold precursor can provide effective evidence in the used strategy.

Characterizations: Investigation of the samples by scanning electron microscopy was carried out using a scanning electron microscope Merlin (Carl Zeiss, Oberkochen, Germany). This microscope is equipped by Gemini column with Schottky field emission cathode as an electron source. The beam stability of the electron source is about 0.2% per hour and 0.4% per day. Spatial resolution for the electron column is stated as follows: 1.0 nm at 15 kV and 1.9 nm at 1 kV. The accelerating voltages range from 0.1 to 30 kV.

Surface morphology was visualized with spatial resolution of 1.5 nm using secondary electron detectors: (i) Everhart Thornley detector (Carl Zeiss, Oberkochen, Germany) and (ii) in-lens semiconductor detector (Carl Zeiss, Oberkochen, Germany). The samples were coated by 3 nm Au/Pd layers using magnetron-sputtering (Quorum, Q150T, Laughton, East Sussex, UK) to prevent the surface charging under the action of the electron beam.

Absorption spectra were collected using UVISIL spectroscopy (HORIBA Jobin Yvon SAS, Longjumeau, France) ellispsometry. Ellipsometric angles (ψ and ∆) are measured by a Xenon lamp (HORIBA Jobin Yvon SAS, Longjumeau, France) (spectral range from 250 to 2100 nm) in air at room temperature with an incident angle of 60° [26]. The absorption spectra are determined by modelling based on two classical Lorentz oscillators .

Atomic force microscopy (AFM) scans were carried out using the Nano-R2TM AFM instrument (NT-MDT Spectrum Instruments, Moscow, Russia) (silicon tips with a spring constant of 0.1–0.6 N/m, cantilever length 450 µm) operated in contact mode in the air at a 0.45–0.75 Hz scan rate and 0° scan angle.

SERS analysis: Raman and SERS measurements were conducted using Dilor Jobin-Yvon Spex instrument from HORIBA (HORIBA Jobin Yvon SAS, Longjumeau, France) with a 632.8 nm laser and CCD detection according to the usual procedures [26].

## 3. Results and Discussion

### 3.1. Shape Control through Altering the Synthesis Parameters

In previous works, [26,27,28,29] we focused on the understanding of PMMA self-assembly via VIPS in order to fabricate MNPs. Akil et al. (2012) [27] revealed the role of the parameters in controlling the size and gaps between silver NP using VIPS. It was concluded that the silver precursor concentration, substrate chemistry, and the thermal treatment of the substrate are the main factors which rule the synthesis process. Then, Kanafer et al. (2016) [28] reported a more advanced mechanistic study of the same method. The authors varied a wide number of physico-chemical parameters including the PMMA solvent (nature and quantity), the non-solvent (nature and quantity), the spin-coating speed, the metal precursor type, etc. Based on this thermodynamic work, a mechanism was proposed, which could be briefly discussed in the following manner. During the spreading of the PMMA mixture on the surface, the repulsive interaction between the couple non-solvent/metallic salt and the PMMA chains results in a micro-phase separation. This process manifests itself through the appearance of vesicles containing the couple non-solvent/metal precursor, which are then immobilized on substrate surface after the complete evaporation of the PMMA solvent. Subsequently, the explosion of the vesicles will happen to allow the evaporation of the non-solvent so that PMMA rings containing metallic salts will be produced. The strong concentration of the metallic precursor on the outline of the PMMA due to the interaction with the esters groups of PMMA results in the formation of MNPs inside the PMMA holes. This study of various parameters and their influence on the nanostructuring process provided us with a good understanding relative to the mechanism of the self-assembly. However, a lack of knowledge about the control of shape using VIPS was still a challenge since we were mainly able to produce nanorings and spherical NPs via this synthesis process. From there, Omar et al. (2017) [26] showed, for the first time, the possibility to fabricate non-spherical NP and particular gold nanocubes with tunable size and morphology by adjusting the gold precursor concentration in the whole PMMA mixture. However, no other shape was shown in this work and the study was limited to the fabrication of monodisperse nanocubes. Based on our understanding from the above mentioned works, we try to extend the method to the synthesis of anisotropic shape of NPs here. It is worthy to note that the only parameter or component of the mixture that we did not vary or modify until now is the metallic precursor type. Knowing that the chemistry and the surrounding medium of the metal atoms play an important role in the growth mechanism and inspired from the role of additives (salts, gaz, and organic solvents), we change the gold precursor molecule. As a result, during the growth of gold NPs on the substrate surface, the seeds will be surrounded by metal salts (M^+^) that were already present in the precursor molecule. It is expected that the interaction of M^+^ with gold seeds stabilizes a given facet, which drives the growth in a preferential and gives rise to a specific morphology. It is interesting to note that the reduction in Au^3+^ and, subsequently, the growth occurs spontaneously upon spin-coating in the liquid medium before the complete evaporation of solvents. Then, the growth will continue until it reaches a stable shape.

According to the above mentioned works, the solvent evaporation rate plays a crucial role in VIPS product [26,27,28]. For this reason, before changing the precursor molecule type, we studied the influence of solvent and non-solvent nature on the morphology of GNPs because the influence of solvent was not investigated. We were interested by the silver as mentioned above. Indeed, depending on the couple solvent/non-solvent used, each PMMA/gold precursor dispersion has its own volatility, which corresponds to the sum of volatility values of all solvents of mixture. From there, the rate of both the reduction in Au^3+^ and the growth of GNPs might be tailored by changing the mixture solvents.

**Figure 1:**
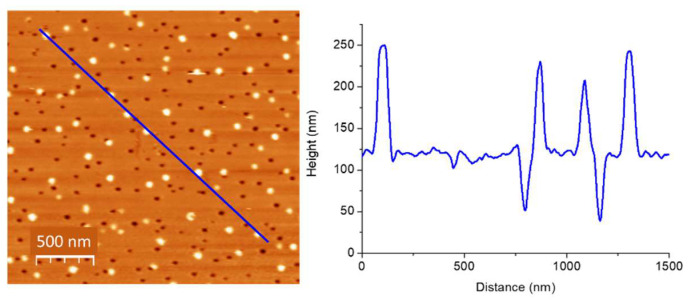
AFM image and its profile for gold nanoparticles embedded in PMMA nanoporous layer using 100 mM of NaAuCl_4_·2H_2_O dispersed in a mixture of ethanol, acetone, and PMMA.

Generally, VIPS provides the formation of GNPs stabilized by PMMA nanoholes as can be observed in Figure 1. A good proportion of these nanoholes is filled with gold nanostructures. According to the AFM scan and corresponding profile, a size control seems to be achieved. More precisely, the minima and maxima have the same amplitude and width, which indicates that GNPs size can be tuned by the PMMA holes size. With respect to the number of nanoparticles, we previously demonstrated that the number is strongly dependent on gold precursor concentration. [16] However, shape control is still not established by VIPS. As assessed above, we recently showed the possibility to fabricate non-spherical GNPs [26].

**Figure 2:**
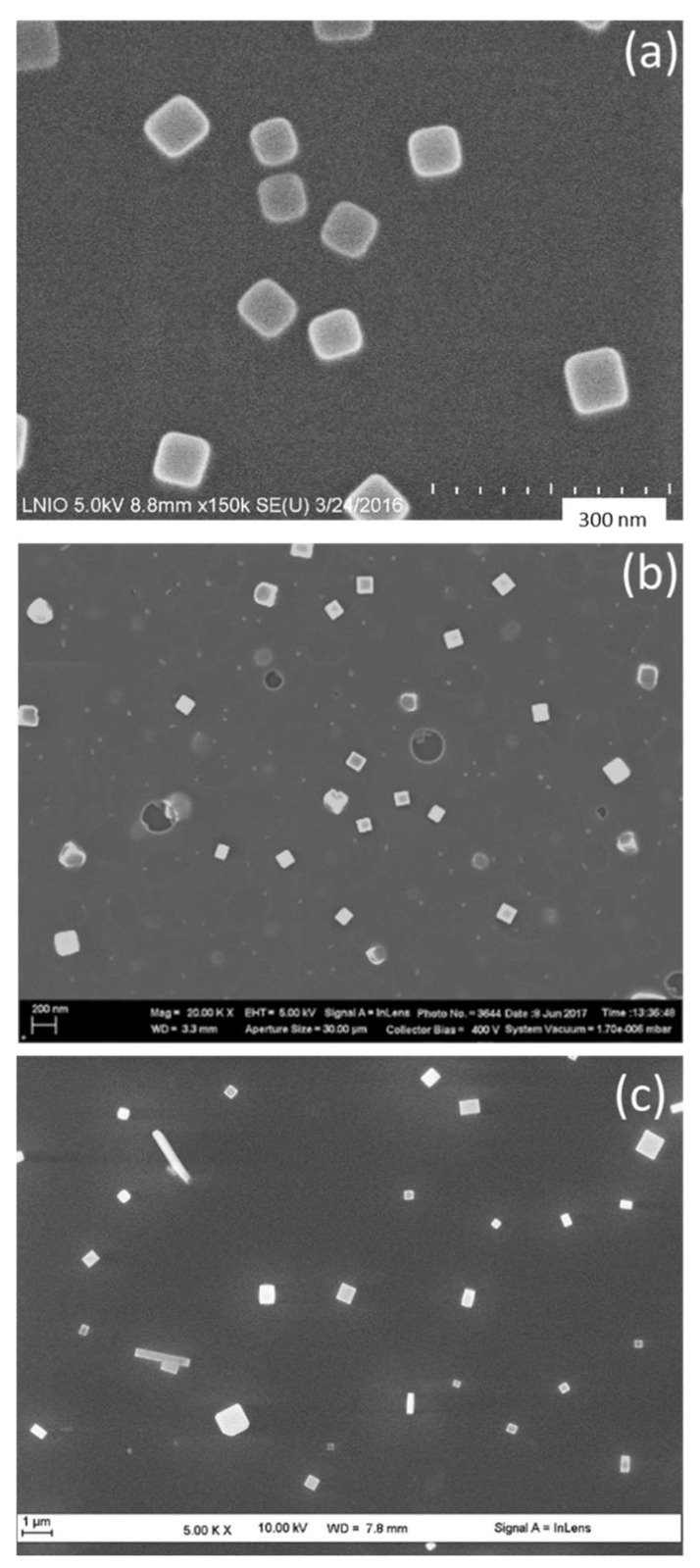
SEM images for Gold nanoparticles obtained with NaAuCl_4_·2H_2_O precursor and various solvent/non-solvent compositions. (**a**) Acetone/ethanol, (**b**) MIBK/ethanol, and (**c**) acetone/isopropanol.

In particular, in order to produce gold nanocubes that require fast kinetics, we used highly volatile solvents (acetone and ethanol), which evaporate rapidly and accelerates the growth rate of NPs as previously established [26]. To fabricate another shape, we tried to change the kinetics rate of formation of GNPs. Starting from this point, we replaced acetone by methyl iso-butyl ketone (MIBK). The samples in Figure 2 were prepared using the same spin-coating parameters (speed 5000 rpm) and NaAuCl_4_ as gold precursor molecule. As a result of changing the PMMA solvent from acetone (Figure 2a) to MIBK (Figure 2b) and the PMMA non-solvent from ethanol (Figure 2b) to isopropanol (Figure 2c), the volatility of the whole dispersion decreases continuously. Consequently, larger gold nanocubes were obtained (Figure 2b). A successive volatility decrease results in the formation of polydisperse nanocubes and rectangles (Figure 2c).

Following the solvent/non-solvent adjustment, we concluded that acetone/ethanol or MIBK/ethanol combination is suitable obtain a single shape, which is cubic using NaAuCl_4_ as the precursor. In particular, to obtain monodisperse GNC, the acetone/ethanol combination is required. Moreover, the formation of rectangular shapes (Figure 2c) might be attributed to the formation of PMMA micelles of different sizes because of the slow evaporation rate of MIBK that results in the aggregation of some PMMA micelles on the silicon surface. An excess of Au^3+^ in these micelles could provide additional growth of gold nanocrystals in one direction, which yields the formation of GNRs [29]. However, the concentration of gold precursor is not enough to form so many rectangles. In this case, further works on tuning Au^3+^ concentration and using a third solvent for PMMA are needed to control the formation of monodispersed GNRs. In the case of MIBK, one can see the formation of larger size of GNP with respect to the formation of bigger micelles of PMMA. This is linked to the slow evaporation rate of MIBK solvent as described above. Furthermore, MIBK results in the production of sharper nanocubes in comparison to those formed with acetone. This outcome can be attributed to the slower rate of nucleation and growth of the nanocubes that is derived from slow evaporation of MIBK, which gives rise to the generation of more crystallized nanostructures (Figure 2b).

**Figure 3:**
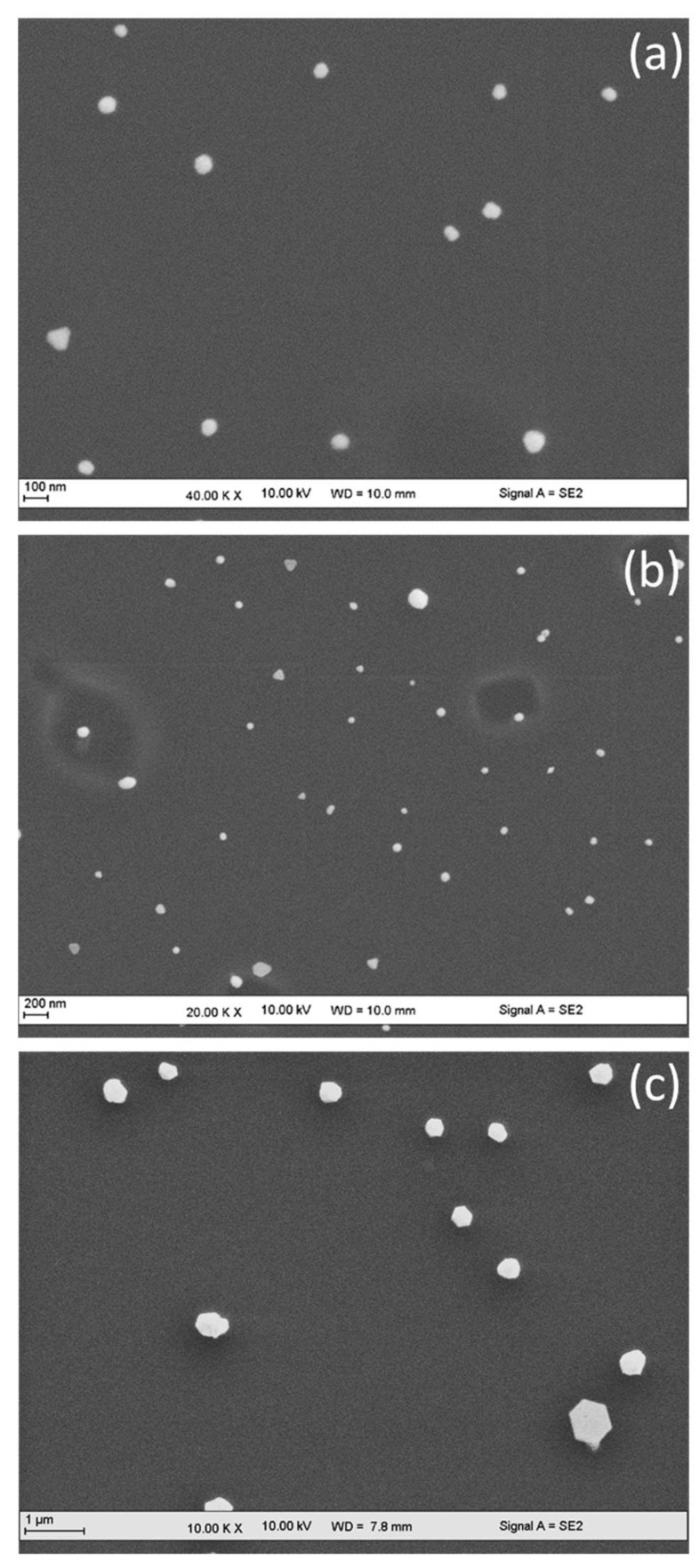
SEM images for gold nanoparticles obtained with HAuCl_4_·2H_2_O (Hydrogen tetrachloroaurate) precursor and various solvent/non-solvent compositions. (**a**) Acetone/ethanol, (**b**) MIBK/ethanol, and (**c**) acetone/isopropanol.

Moreover, the role of PMMA solvent was studied using another gold precursor molecule (chloroauric acid, HAuCl_4_). One can see in Figure 3 the same behavior observed in Figure 2. Mainly, the hexagonal gold nanoparticles (GNH) were obtained when NaAuCl_4_ was replaced by HAuCl_4_. This latter molecule should decrease the pH of the PMMA/precursor dispersion. Through spin-coating, it permits the reduction in a low number of Au^3+^, thus resulting in slower nucleation and growth. This kinetically slow mechanism resulted in the formation of GNH instead of GNC since the hexagonal morphology requires slower reaction rate. Similarly, as described before (Figure 2), lower evaporation rate of MIBK induces the formation of polydisperse and mainly larger NPs. It is worthy to note that some gold nanotriangles are observed with MIBK solvents (Figure 3b), which can be related to an excess of Au^3+^ ions existing in some PMMA micelles on the surface of silicon substrate. In this context and through the SMG method, it was possible to transform hexagons or octahedrons into nanotriangles by increasing the gold precursor concentration until a specified value [17,18,19,20,21].

From this section, we demonstrated that the NaAuCl_4_/acetone/ethanol combination generates monodispersed nanocubes, whereas the HAuCl_4_/acetone/ethanol combination generates nanohexagons.

Indeed, changing the PMMA solvent was not crucial for shape control. Nevertheless, it was helpful for controlling the size, number, and gaps between the fabricated gold nanoparticles.

### 3.2. Controlling the Shape of GNP via Altering the Gold Precursor Molecule

Based on the above experiments, we visibly demonstrated the interest to change the precursor type (molecule) on the shape of GNP. No matter solvent/non-solvent combinations were used, the precursor molecule was the main component that defined the shape. It was shown that NaAuCl_4_ generates nanocubes and HAuCl_4_ mainly generates hexagons (or octahedrons). Until now, we achieved the control (size and shape) of nanocubes fabrication (using ethanol-PMMA/Acetone). Still, further studies are required to achieve the formation of monodispersed nanohexagons. We also need to explore the possibility to produce other morphologies. Here, we investigate the role of the gold precursor (pH and counter-ion size, etc.) on the synthesis products.

**Figure 4:**
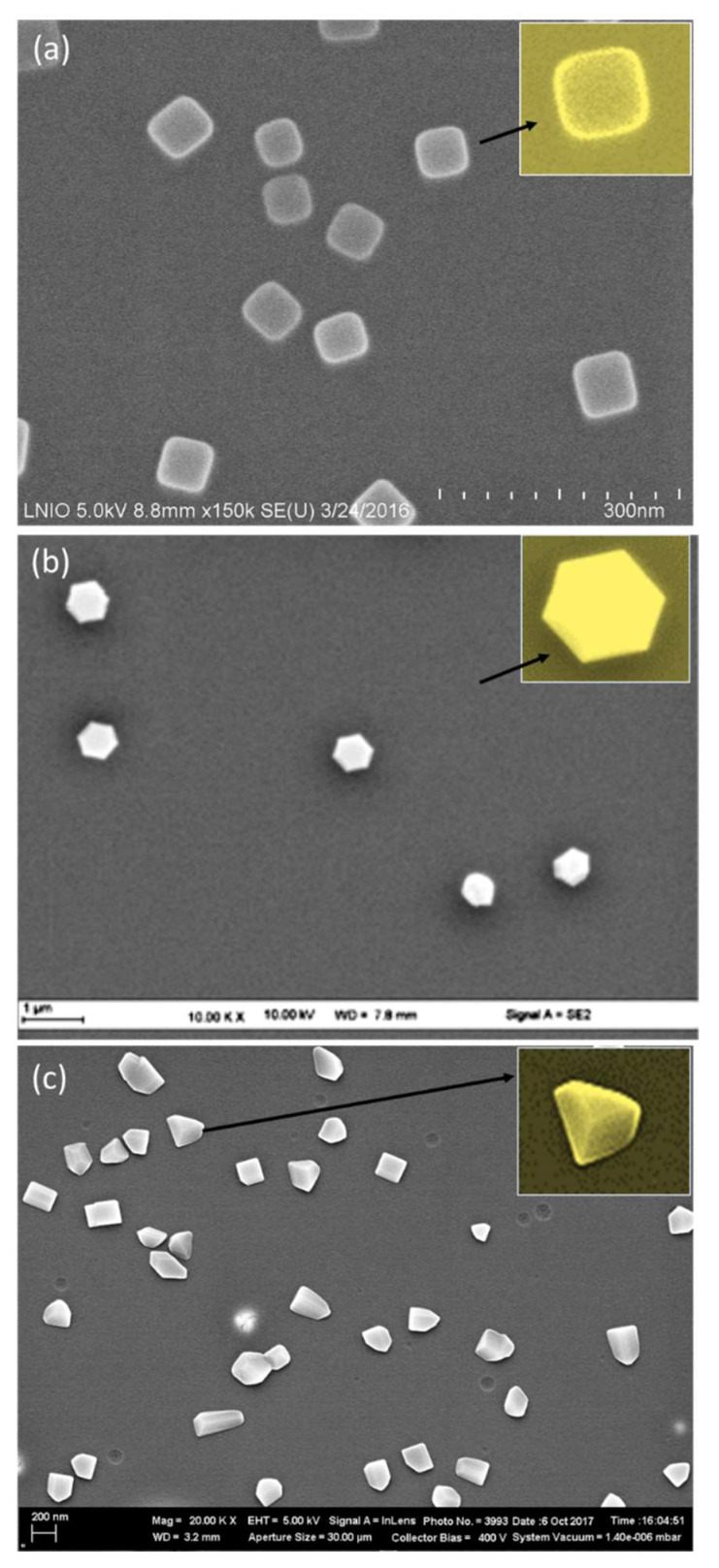
SEM images for three samples of gold nanoparticles prepared with a combination of acetone (PMMA solvent) and isopropanol/ethanol (precursor solvent), where ethanol was used in (**a**) and isopropanol in (**b**) and (**c**). The samples basically differ in the gold precursor molecules: (**a**) 60 mM of NaAuCl_4_, (**b**) 100 mM HAuCl_4_, and (**c**) 100 mM KAuCl_4_. The inserts in (**a**), (**b**), and (**c**) show the formation of monodispersed nanocubes, nanohexagons, and nanoprisms, respectively. Scale bars are 200 nm for a and (**c**) and 1 µm for (**b**).

Aiming to fabricate uniform triangular and hexagonal shape, we changed the precursor molecule and we selected the acetone/isopropanol combination for two reasons. First, acetone is suitable for low polydispersity. Second, isopropanol generated more hexagonal shapes Figure 3c) than ethanol and it has lower volatility, giving rise to triangular nanostructures that require low growth rate of formation. Figure 4c reveals the formation of nanoprisms when HAuCl_4_ is replaced by KAuCl_4_. This result confirms once again that the precursor molecule plays the major role in controlling the shape. The used three precursors differ by their molecular weight and their counter-ion (Na^+^, H^+^, and K^+^) of AuCl_4_^−^. We propose that the counter-ion plays an important role in the synthesis mechanism. It could be compared to the role of additives used for controlling the shape of NPs in SMG methods. For example, Ag^+^ has been used in seed-mediated methods to stabilize high-index facets through under potential deposition mechanism (UPD) [30,31]. Figure 4a shows gold nanocubes using NaAuCl_4_. However, gold nanoprisms were obtained using KAuCl_4_ (Figure 4c).

It is worth noting that the formation of GNPs occurs on the substrate and, as assessed before, the reduction onset arises before the complete evaporation of the solvents. These solvents are organic and the main part is non-polar (MIBK, acetone). A wide number of works reported strategies for shaping the control of NPs, especially gold nanomaterials in water. However, surface-based strategies such as the process described in the present study, have not been widely investigated [30]. 

To date, all the relevant synthetic methods in non-aqueous medium correspond to the polyol process [31,32,33]. Among the wide number of synthetic methods of anisotropic GNPs in literature, we were especially interested by the polyol process. This is because of the similarities in the reaction medium used, including the non-polar solvent and the presence of polymer as compared to PMMA in our approach. The term “polyol process” was first used in the late eighties as a liquid-phase synthesis route to obtain finely divided metals from their oxides, hydroxides, or salts in polyalcohols. The basic concept was to prepare metal powders using a liquid organic compound acting both as a solvent of the solid precursor and as a reducing agent. Polyalcohols such as α-diols and ether glycols, resulting from their condensation appeared very convenient for this purpose [22]. Polyol synthesis provides the formation of GNPs through a thermal reduction in a gold precursor in an organic solvent such as N,N-dimethylformamide (DMF) using usually poly(vinyl pyrrolidone) as the stabilizer. Although the development of this process occured through altering the chemistry of the synthesis medium, good control of shape is still not achieved in the whole solution [23,24,25].

However, we can summarize the main methods of allowing shape control in the polyol method as the following:

- The interaction of PVP with particular crystal facets is crucial in determining the shape of GNPs [34].
- The crystalline structure of the seeds definitely influences the growth. Twinned and single crystalline seeds result in different shapes of NPs [35].
- The selective underpotential deposition (UPD) of Ag^+^ onto {100} gold facets rules the final shape through selective surface overgrowth through the suppression of the epitaxial overgrowth of additional gold layers [36].
- The addition of small amounts of different salts can strongly influence the final particle shape through preferential adsorption on specific gold facets or changes in their relative surface energy [37].

Based on this latter point, Na^+^, K^+^, and H^+^ amounts that contains the gold precursor in our case may strongly affect the final shape of GNPs. Both the size and the pH of each ion involve selective adsorption on certain facet of gold seeds, which results in selective surface growth and, finally, the production of the specific shape. This suggested hypothesis could be reinforced by some experimental points: all the experimental conditions governing VIPS (solvents and their amounts, spin-coating parameters, substrate surface, and amount of gold precursor) were fixed. Only the gold salt type was changed, keeping its amount stable in the three mixtures used to produce three different shapes of GNPs.

Kim et al. (2004) showed that small amounts of silver ions (1% of the gold precursor) induced the formation of uniform gold nanocubes through a one-pot polyol process. The authors also emphasized the influence of the salt amount on the final shape.

It was demonstrated that salt additives are responsible for shape-direction by affecting preferential attachment fluxes of Au atoms to different surfaces during growth, as reported previously [31,32,33,34,35,36,37,38]. Nevertheless, the number and type of additives identified until now for shape control remain very limited, which suggests that chemical interactions are important. For MNPs, the interplay of halide ions (Br^−^, Cl^−^, and I^−^) is critical and a general mechanism based on simple facet blocking does not explain many features of anisotropic particle growth [39]. On this basis, the formation of gold nanoprisms and nanohexagons that have octahedral morphologies can be attributed to the preferential adsorption of K^+^ or H^+^ on the {111} planes of Au nuclei that inhibit the growth rate along the {111} direction suitable to produce triangular and octahedral geometries. H^+^ may move faster with respect to the corresponding selective planes because it is smaller than K^+^, resulting in the rapid growth rate on {111}, which favors the formation of hexagons rather than triangles or prisms. It is well established that the surface energy of {100} plane is higher than that of {111} plane, in relation to the higher chemical reactivity [31,39]. Generally, chemisorption is favored for smaller atoms or molecules. In this context, due to its smaller size the attachment fluxes of Na^+^ might be higher than K^+^. Therefore, Na^+^ will favor the growth along {100} planes, which is suitable for the formation of cubic shapes. The recent observation by Omar et al. [26] (2017) showed that the amount of the precursor molecule (i.e., the quantity of Na^+^) does not influence the morphology; on the other hand, changing the molecule precursor type in the present paper results in the production of cubic ({001} bound) and octahedral ({111} bound) morphologies and supports the hypothesis that counterions could be the shape-direction agents in the VIPS process. This mechanistic discussion remains a hypothesis, in concert with much research on the shape control of NPs and with the experimentally-supported mechanism.

Moreover, in comparison with the conventional surfactant-based protocols which changes the product of the synthesis by varying the reaction rate, the obtained nanostructures cannot be explained in terms of those proposed for surfactant-based growth modes where surfactants, surface diffusion, and/or collision patterns are used to transform the reaction product. However, we propose a nucleation and growth pathway that relies on the formation of a space charge region around each seed consisting of a layer of ions (Na^+^ or K^+^), where the whole layer is dependent on the facets expressed by the seed, the rate at which the reduced ions are being deposited, and the pH of the solution. This work implies a rich nature of surfactant-free synthesis as well as the utility of the substrate-based platform in defining the growth strategies. According to all the obtained results, we are interested in determining the plasmonic and sensing properties of monodispersed nanocubes, nanohexagons, and nanoprisms.

**Figure 5:**
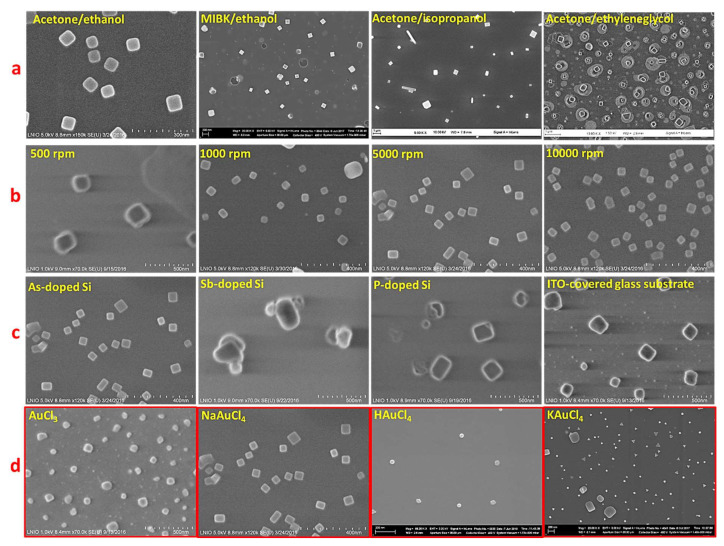
SEM images of GNP obtained under various experimental conditions. (**a**) NaAuCl_4_/PMMA/different solvent couples using 5000 rpm as spin coating speed and As-doped silicon substrate. (**b**) NaAuCl_4_/PMMA/acetone/ethanol using As-doped silicon substrate and various spin coating speeds. (**c**) NaAuCl_4_/PMMA/acetone/ethanol using 5000 rpm as the spin coating speed and various substrate conductivities. (**d**) NaAuCl_4_ or HAuCl_4_ or KAuCl_4_/PMMA/acetone/ethanol using 5000 rpm as spin coating speed and As-doped silicon substrate.

As a summary of the mechanistic study that has been conducted, we show in Figure 5 the influence of the parameters that rules the VIPS methods. Thus, we varied the solvent/non solvent couple, the spin coating speed, the surface conductivity, and the precursor molecule. Only the latter parameter results in the formation of various shapes of GNPs, which highlights the crucial role of counter ions in driving the growth mechanism.

More precisely, in Figure 5 we fixed the precursor molecule to NaAuCl_4_. In this case, gold nanocubes were mainly obtained and the solvents, spin-coating speed, and surface conductivity influenced the size and gaps between the NPs. However, when we change the precursor type, we observed the morphology change. To highlight this finding that is only related to the precursor type, we colored the outline of Figure 5d in red. On this basis, once the precursor molecule is fixed, it was necessary to adjust afterwards the mechanism kinetics through the solvent/non-solvent couples to obtain a precise and selective control of morphology as observed in the different synthesis mechanistic methods illustrated in Figure 2, Figure 3 and Figure 4.

### 3.3. SERS Enhancement as a Function of GNP Shape

**Figure 6:**
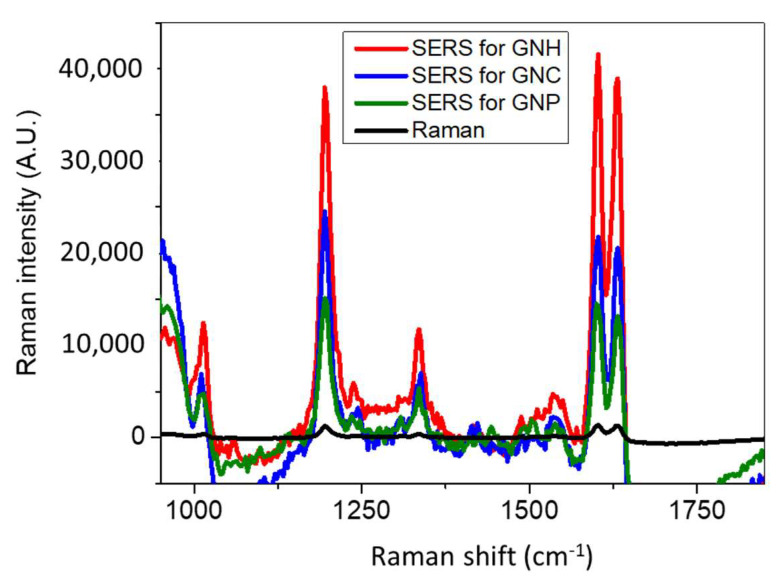
SERS spectra of 10^−12^ M of BPE probed with three different shapes of GNP. Raman measurements were obtained with 10^−2^ M of BPE. Spectra were obtained with a 632.8 nm laser at P = 10 mW and acquisition time = 10 s. Each curve is an average of 10 spectra collected from different positions on the substrate.

As assessed before, anisotropic MNPs often display better optical properties compared to spherical ones, in part due to the intense electric field localization near sharp geometric features and a broadly tunable localized surface plasmon resonance [26]. As a result, non-spherical NPs are attractive building blocks for SERS substrates. To make these blocks potential as SERS sensors, one should be able to (i) generate a sufficient number of SERS hotspots with structures of controlled shape and size and (ii) remove ligands so that analytes can easily access NP surface sites. This latter feature is already acquired in our case since the synthesis method is ligand-free. In this context, in aiming to perform SERS measurements on the substrates described above and to study the influence of shape on the sensing features, we tried to optimize the synthesis in order to obtain substrate with high number of GNPs of similar size and density on the surface. Fixing the structural properties and distance between GNPs was necessary to investigate the relationship between morphology and SERS enhancement. To make this possible, the spin-coating parameters and the gold precursor concentration were tuned to obtain samples of comparable size distribution. As a result, we obtained the three samples illustrated in Figure 4 and performed SERS for Bipyridine ethylene (BPE) as shown in Figure 6.

**Figure 7:**
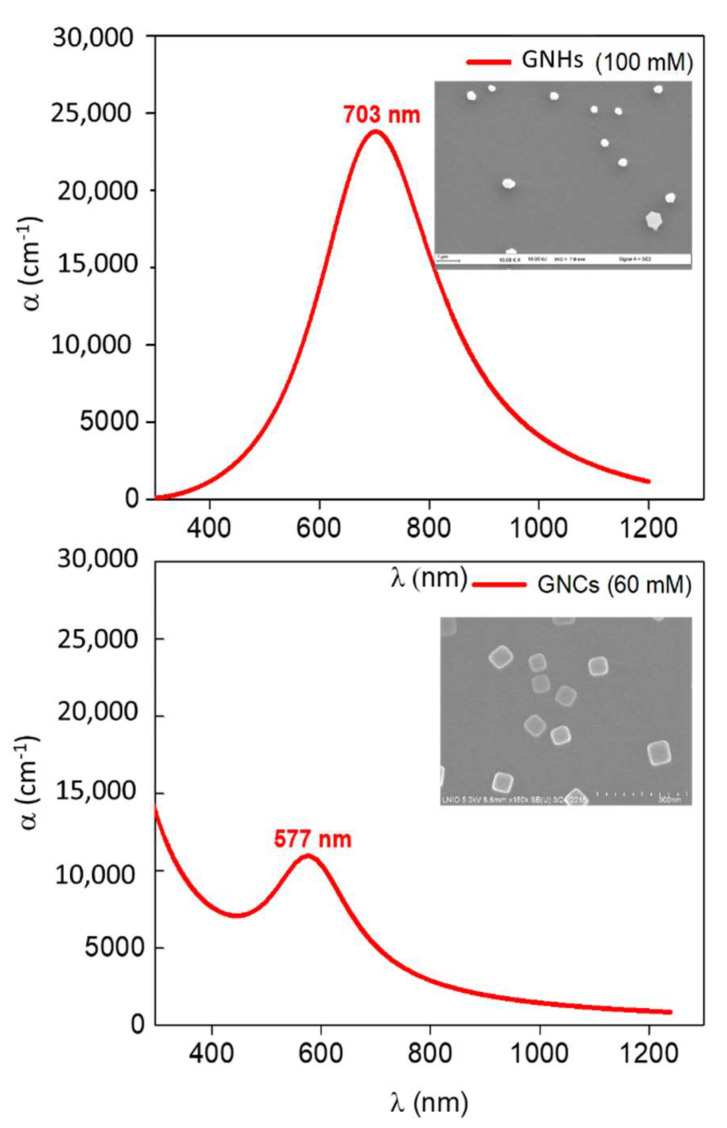
Absorption coefficient spectra for gold nanocubes prepared with 60 mM of NaAuCl_4_ precursor and for GNH prepared using 100 mM of HAuCl_4_ precursor. The inserts correspond to Figure 4a,b, respectively.

For a better understanding of the SERS results, we used ellipsometric spectroscopy to determine the plasmon resonance band of samples. The two Lorentz oscillators based model were described in a previous work [26]. The corresponding absorption coefficient spectra of these samples are illustrated in Figure 7.

A redshift was observed in the maximum absorption position (plasmon band) from 577 nm for GNC to 703 nm for GNH. This significant shift might be mainly due to the bigger size of GNH. The size of GNC is ~65 nm and GNH size is ~250 nm. The surprising broadness of the GNH plasmon band can be attributed to the PMMA nanoholes contouring GNP, which contributes to the reflection signal detected and cannot be considered by ellipsometric measurements.

In contrast, it was impossible to model the ellipsometric measurements and determine the plasmon band of the nanoprism substrate (Figure 4c) due to the high thickness of PMMA (~300 nm) that does not fit the limits of the used model.

Although the GNC possess a plasmon band position (577 nm) that matches the Raman excitation wavelength (632.8 nm), GNH exhibits the best Raman enhancement. Moreover, GNC revealed higher sensitivity than nanoprisms in relation with lower SERS intensities, especially for the most intense peak at 1605 cm^−1^.

The SERS enhancement factor for the three substrates SEF was determined following a procedure established in a previous study [26]. We employed the Raman band of 1605 cm^−1^ to calculate the SEF through the following equation:

SEF = (I_SERS_ × N_normal_)/(I_normal_ × N_SERS_) (1)

where I_SERS_ and I_normal_ correspond to the intensities of 1605 cm^−1^ band for both SERS and normal Raman spectra, N_normal_ is the number of molecules probed for a normal Raman setting, and N_SERS_ is the number of molecules detected in SERS.

N_normal_ and N_SERS_ values were calculated by taking into account the excited NPs present in the surface area of the laser spot, in relation with the samples SEM images. Indeed, each NP was considered as a half-sphere in this calculation. First, we estimated the number of BPE molecules that can be adsorbed on each hemisphere by considering the size of BPE molecule and the angle that forms with the substrate upon adsorption through the nitrogen atom. In the excited surface, we determined the number of hemispheres and subsequently the number of BPE molecules. This procedure was adapted for both SERS and Raman signals. N_normal_ was determined based on the Raman spectrum of a 10^−2^ M of BPE. The focal volume of the used Raman system is 0.62 fl. In this volume, we detected 3 × 10^6^ molecules in Raman and 40 molecules in SERS for 10^−12^ M of BPE. On this basis, the SEF for GNH reached ~10^12^ against 2 × 10^9^ for GNC and 10^9^ for GNP. These values match the highest SEF value (10^14^) found for BPE in the literature and highlights the ultrasensitivity of the produced substrates [15,16,26]. We can explain this sensitivity trend as a function of the shape by the electric field concentrated at the corners of GNP. Anisotropic MNPs show field increases that relates to the enhanced localization of charge density at a tip of the NP. When this tip is excited by an electromagnetic field, a strong electric field appears in these NP regions, resulting in large field enhancement localized at the tips [40,41,42]. Thus, the high surface energy area of anisotropic NPs influences its chemical reactivity. It has been found that the rate of photochemical reaction of the molecules adsorbed on GNPs can be controlled by their surface geometry [43]. Thus, the more angles are on NP the higher the sensitivity. As a result, the higher sensitivity of GNH (blue curve) in comparison to GNC (red curve) is due to the presence of the higher number of corners and then of hot spots on the whole substrate, which is relevant for SERS. Moreover, the large size of the GNH deals with the large surface of interaction between the target molecule and the NP, which permits higher coverage of the substrate by the analyte and results in higher SERS intensity. Such aspects about the effect of morphology on the sensing properties were widely investigated by other groups. Nevertheless, most studies did not allow a good understanding about the impact of morphology on sensitivity because colloidal NPs are usually used in detection due to their ultrasensitivity [13,14,44]. However, a good control of such systems in terms of size, surface roughness, interparticle distances, and shape is generally difficult [26,45,46].

As a perspective, in order to better elucidate the effects of shape on the sensitivity of the obtained samples, a better control of GNPs size and the gaps between them is required. It is worthy to note that the studied substrates were not the most suitable for SERS since we only focused on the influence of morphology on the sensitivity. In all samples, the NPs were separated by large gaps. An optimization of synthesis could allow this condition to be easily achieved based on our previous studies [26,27,28,41].

**Figure 8:**
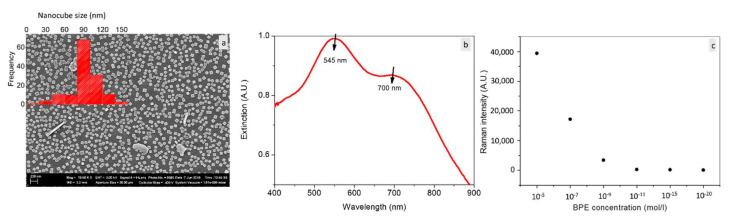
(**a**) SEM images for gold nanocubes prepared with 60 mM of NaAuCl_4_/ethanol/acetone combination upon spin-coating on ITO-covered glass substrate. The insert is a size distribution of the corresponding nanocubes performed by imageJ. (**b**) Extinction spectrum obtained for the GNC obtained in (a). (**c**) SERS intensity of the 1600 cm^−1^ band as a function of BPE concentration obtained for the same substrate over 0.5 s.

Herein, we show in Figure 8 a huge increase in the number of gold nanocubes of Figure 4a when the synthesis was performed on a more conductive substrate (glass wafer covered with ITO thin layer).

Knowing that the substrate due to its conductive characteristics induces the formation of GNPs, their density is thus strongly substrate dependent. More of NPs can be thus produced with ITO than silicon. Therefore, more nanocubes were obtained using ITO substrate in relation with the increased available free electrons on the ITO surface allowing spontaneous and strong reductions of increased Au^3+^. As a result, the substrate completely covered by GNC and then by the hotspots was obtained. Sharp and monodisperse GNC were produced using ITO. Moreover, narrow size distribution was obtained and the nanocube size was mainly centered at 90 nm. Interestingly, it was possible for this substrate to only obtain an extinction signal using an experimental optical setup due to the high number of nanocubes on the substrate surface. The extinction measurements showed two plasmonic bands. The 445 nm band is attributed to the optical response of 90 nm GNC according to Omar et al. [26] (2017). The 700 nm band is attributed to the coupling between closely separated GNC. Based on the shown strong interaction between the NPs, which indicates the large number of hotspots, SERS measurements were performed for BPE of lower concentration aiming to detect the single molecule. One can see in Figure 8c the possibility to detect 10^−20^ mol of BPE during 0.5 s, which corresponds to an enhancement factor of 10^17^ and the single molecule of BPE detected. On this basis, we consider 10^−20^ M as the detection threshold of BPE using our samples. This value exceeds the highest value (10^14^) for the SERS enhancement factor published until now. The created substrate could be highly efficient for biodetection, including biomolecules, diseases and pathogens that require rapid, label-free and ultrasensitive sensors. All these features were realized in the synthesized substrates in addition to the reproducibility.

**Figure 9:**
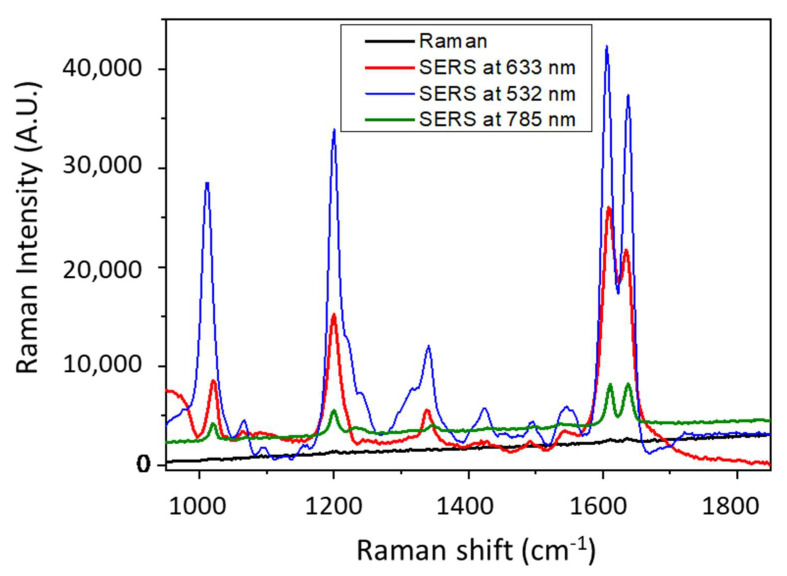
SERS spectra of 10^−20^ M of BPE probed at different wavelength for the sample shown in Figure 8. Raman measurements were obtained with 10^−2^ M of BPE. Spectra were obtained at P = 10 mW over 10 s. Each curve is an average of 10 spectra collected from different positions on the substrate.

Knowing that the GNC present a dominant band centered at ~545 nm as observed in Figure 8b, we performed SERS on these nanocubes at different laser wavelength. Figure 9 shows an increase in SERS intensity when the excitation wavelength decreases and reaches a maximum at 532 nm.

This latter matches the plasmonic band (545 nm) of GNC and results in higher SERS intensity. Basically, the excitation energy plays a crucial role in SERS enhancement and must be the closer to the surface plasmon resonance peak [26]. From there, in a future study, we aim to produce other morphologies and to further investigate the sensing properties using other probe molecules and photonic parameters including laser wavelength, and lower acquisition time.

**Figure 10:**
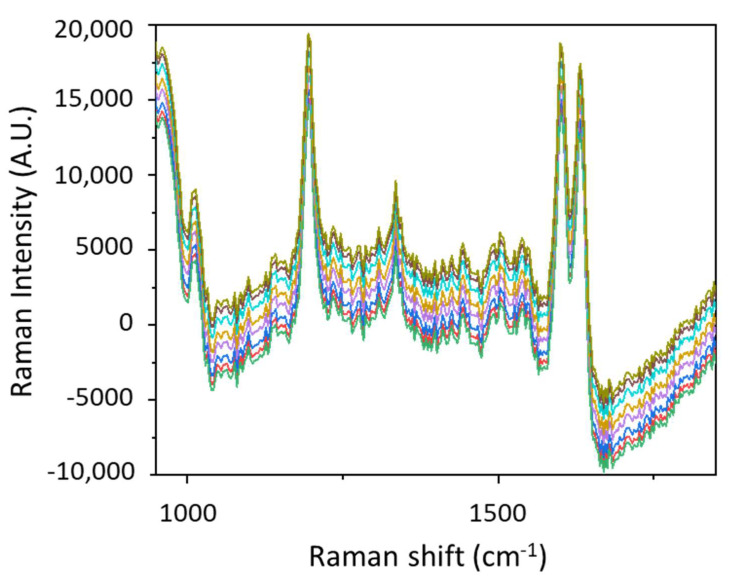
SERS spectra of 10^−20^ M of BPE probed at different zones on the substrate with a time interval of 1 month for the sample shown in Figure 8. Raman measurements were obtained with 10^−2^ M of BPE. Spectra were obtained at P = 10 mW over 5 s.

We observed that despite the randomly distributed hot spots due to the variation of gap distances between GNPs, SERS results are reproducible for a given substrate and for several similar substrates. To obtain a better idea about this reproducibility, we illustrate in Figure 10 the SERS signal of 10^−20^ M performed at different times on the same substrate with an interval of 1 month. Each SERS spectrum was obtained on another spot on the substrate and repeated five times. A remarkably small intensity variation highlights a reproducibility of ~95%, which could be the lowest among the previously reported substrates fabricated by various techniques. [13,14,15,16,17,18] These findings are promising, especially for sensing since it is always challenging to have a SERS substrate with adequate signal enhancement and lower intensity variation.

## 4. Conclusions

We have demonstrated the shape-controlled growth of gold nanostructures by a substrate-strategy. We found that varying the reaction kinetics allowed different growth modes of GNPs. In this manner, the silicon electrons bounded to the counter ions (H^+^, Na^+^, and K^+^) of the gold precursor molecule used and serve as heterogeneous nucleation for gold nanocrystals. These anisotropies on the surface of silicon result in anisotropies in the kinetic growth associated with the following: (i) Au atoms arriving preferentially to the base of nucleation site from an adjacent collection area, (ii) higher atom diffusion rates on {111} facets than on {100} ones, and (iii) the various Ehrlich–Schwoebel barriers, which inhibit the atom motion between facets. These anisotropies result in the non-equilibrium steady state strongly dependent on whether the reaction is carried out in a regime of slow, moderate, or fast kinetics. Slow kinetics achieved by the KAuCl_4_ combination gave rise to triangular gold nanoprism generated through epitaxial deposition on just a small portion of a single {100} Au facet followed by the propagation of a rapid growth front away from the seed. Moderate kinetics permitted by HAuCl_4_ resulted in hexagonal geometry realized through layer-by-layer deposition of Au on all {100} equivalent seed facets followed by the overgrowth of all remaining facets. Fast kinetics allowed by NaAuCl_4_ produced cubic and rectangular morphologies, where the overgrowth more closely follows the topography of the underlying seed. The so-formed substrates present the opportunity to form versatile devices for catalysis, sensing, and photovoltaic applications. The detection of the molecule of BPE through SERS revealed a strong relationship between the substrate sensitivity and GNP shape. The more corners in the nanoparticle, the greater the sensitivity. Thus, the SERS enhancement increased while going from the prisms towards the cubes then towards the hexagons, which is related to the increase in hotspots density by NP surface area.

## Data Availability

The data presented in this study are available on request from the corresponding author.
